# Stem Cells Derived from Neonatal Mouse Kidney Generate Functional Proximal Tubule-Like Cells and Integrate into Developing Nephrons *In Vitro*

**DOI:** 10.1371/journal.pone.0062953

**Published:** 2013-05-07

**Authors:** Egon Ranghini, Cristina Fuente Mora, David Edgar, Simon E. Kenny, Patricia Murray, Bettina Wilm

**Affiliations:** 1 Institute of Translational Medicine, Faculty of Health and Life Sciences, The University of Liverpool, Liverpool, United Kingdom; 2 Department of Paediatric Surgery and Urology, Alder Hey Children’s NHS Trust, Liverpool, United Kingdom; Muséum National d'Histoire Naturelle, France

## Abstract

We have recently shown that kidney-derived stem cells (KSCs) isolated from the mouse newborn kidney differentiate into a range of kidney-specific cell types. However, the functionality and integration capacity of these mouse KSCs remain unknown. Therefore, the main objectives of this study were (1) to determine if proximal tubule-like cells, generated *in vitro* from KSCs, displayed absorptive function typical of proximal tubule cells *in vivo*, and (2) to establish whether the ability of KSCs to integrate into developing nephrons was comparable with that of metanephric mesenchyme (MM), a transient population of progenitor cells that gives rise to the nephrons during kidney organogenesis. We found that proximal tubule-like cells generated *in vitro* from mouse KSCs displayed megalin-dependent absorptive function. Subsequently, we used a chimeric kidney rudiment culture system to show that the KSCs could generate proximal tubule cells and podocytes that were appropriately located within the developing nephrons. Finally, we compared the ability of KSCs to integrate into developing kidneys *ex vivo* with that of metanephric mesenchyme cells. We found that KSCs integrated into nascent nephrons to a similar extent as metanephric mesenchyme cells while both were excluded from ureteric bud branches. Our analysis of the behavior of the two cell types shows that some, but not all KSC characteristics are similar to those of the MM.

## Introduction

In mammals, the permanent kidney is derived from two mesodermal cell populations: the ureteric bud (UB) that gives rise to the collecting ducts and ureters, and the metanephric mesenchyme (MM) that generates the nephrons. The survival, growth and appropriate differentiation of the UB and MM are regulated by reciprocal signaling between the two populations. Nephron development begins when signals from the UB induce the MM to condense around the UB tips. The MM cells then undergo mesenchymal-to-epithelial transition (MET) to form the renal vesicle, which elongates to form the nephron tubule, comprising the glomerulus, proximal and distal tubules [1]. Prior to induction, the MM expresses low levels of the transcription factors, Pax2 and Wt1, both of which are crucial for kidney development [2], [3], but after the MM has undergone MET, the expression of both proteins is increased [4]. As the renal vesicle elongates, Wt1 becomes highly expressed in the nascent podocytes of the glomeruli, but is downregulated in all other cell types within the developing nephron [5]. Conversely, Pax2 is rapidly downregulated in the nascent podocytes, but continues to be expressed by the remaining cells of the nephron tubule and UB until kidney development is complete [6].

Although the MM is induced to undergo nephron formation *in vivo* by signals emanating from the UB, if MM cells are separated from the UB and cultured *in vitro*, they can be triggered to undergo MET and generate nephron structures by a range of diverse ‘inducers’, including the dorsal spinal cord [7], [8], Wnt-expressing cells [9]–[11], and the glycogen synthase kinase-3 (GSK3) inhibitors, lithium and 6-bromoindirubin-3′-oxime (BIO) [12], [13]. This shows that MM cells are committed to generate cells of the renal lineage, and for this reason, could potentially be used to replace diseased or damaged cells in the ailing kidney. However, MM cells rapidly degenerate following their isolation from the kidney rudiment, and although the addition of Bmp7, and Fgfs +/− heparin [14], [15] or culture on Wnt4-expressing cells [11] can promote survival and cell growth, population expansion is modest and the MM cells only maintain their phenotype for up to two weeks. The limited proliferation capacity of the MM cells *in vitro* is not surprising, for even *in vivo*, the cells undergo a discrete number of cell divisions and are no longer present following the cessation of nephrogenesis [16], probably reflecting the fact that the MM represents a progenitor, rather than a stem cell population. It should be noted that in the current work, the term ‘stem cell’ refers to a cell that displays both long-term self-renewal capacity and the potential to differentiate, whereas ‘progenitor cell’ refers to a cell with differentiation potential, but only a limited capacity to self-renew. The absence of nephron progenitors in the mature mammalian kidney is different from the situation in lower vertebrates, such as the zebrafish, where nephron progenitors persist throughout adulthood [17], and likely explains why the adult fish kidney can generate new nephrons following injury [18], [19] whereas the adult mammalian kidney cannot [16], [20].

Despite the fact that MM cells are not present in the adult mammalian kidney [16], there have been several reports showing that adult rodent and human kidneys contain one or more stem/progenitor cell populations [21]. In rodents, both the renal papilla and proximal tubule appear to contain stem/progenitor cells [22]–[24] and in humans, such cells have been identified in the tubular compartment of the renal cortex, within the Bowman’s capsule, and in the papilla [25]–[31]. Several of the aforementioned studies have shown that some kidney stem/progenitor cells can differentiate into a variety of renal cell types, both *in vitro* and *in* *vivo* [24], [28], [29]. However, in most cases, differentiation was defined solely on the basis of marker analysis and it remains to be established whether these stem/progenitor cell-derived renal cell types display any functionality. Nevertheless, the apparent ability of the stem/progenitor cells to generate renal cell types has led to the suggestion that their role in the adult mammalian kidney is to generate specialized renal cells for normal cell turnover and in response to acute injury [22], [26]. Still, although kidney-derived stem/progenitor cells administered to rodents with induced renal injury can integrate into the kidney and improve renal function [24], [26], [31], evidence suggests that repair following acute tubular injury is mediated by proliferation of surviving tubular cells, rather than by a resident stem/progenitor cell population [16], [32]. Furthermore, a recent report has suggested that human kidney-derived stem/progenitor cells improve renal function by secreting paracrine factors that promote tubular repair [33]. Taken together, these studies raise questions about the normal role of kidney-derived stem/progenitor cells *in vivo*, and the relationship between postnatal kidney stem/progenitor cells and the MM.

We have recently isolated a stem cell population from the mouse kidney that displays unlimited self-renewal, expresses a range of MM markers, including Wt1 and Pax2, and can generate cells *in vitro* that express markers of podocytes and proximal tubule cells [34]. Of note, these kidney-derived stem cells (KSCs) were isolated from neonates, a life-stage where the mouse kidney is still undergoing nephrogenesis [35] and thus contains MM cells. The aims of the current study were to determine if proximal tubule-like cells generated *in vitro* by the neonatal KSCs displayed any functionality, and to investigate if the KSCs could differentiate appropriately in the developing kidney to generate proximal tubule cells and podocytes that were correctly positioned within the nascent nephrons. Furthermore, given that KSCs are derived from the MM and express a range of MM markers [34], we used a kidney rudiment culture system described by Davies and co-workers [36] to investigate whether the KSCs could integrate into developing kidney structures to a similar extent as MM cells.

## Materials and Methods

### Cell Culture

Primary cultures of MM cells were obtained from embryonic day (E) 11.5 CD1 mouse kidney rudiments (Charles River). Metanephroi were incubated in 0.5 mg/ml collagenase type I (Sigma) for 5 min at 37°C, and the MM was then teased away from the UB. MM cells were cultured in DMEM/F12 (Sigma), in the presence of 10% fetal calf serum (FCS) (PAA Laboratories), 2 mM L-glutamine, 1X insulin/transferrin/selenium (ITS), 20 ng/ml dexamethasone, 100 units/ml penicillin, 0.1 units/ml streptomycin (all from Sigma) (MM medium), supplemented with 25 ng/ml Bmp7 (R&D System) and 100 ng/ml Fgf2 (Cell Signaling). The cells were cultured inside a 20 mm silicon chamber, within a tissue culture dish (both from Greiner Bio-One), in a total volume of 600 µl of culture medium and subcultured every 4 days, for a maximum of 12 days. The KSC clonal line, H6, was cultured in high glucose DMEM (Sigma) supplemented with 10% FCS and 2 mM L-glutamine (KSC medium), as previously described [34]. All experiments were carried out using H6 KSCs between passage 10 and 20. The expansion of MM cells was assayed by inoculating 2.5×10^3^ cells (n = 5 or more) in a 96-well plate (Nunc), using Cell Counting Kit-8 (Sigma), following manufacturer’s instructions. Absorbance measurements were taken with a SpectraMax microplate reader (Molecular Devices) at 24 hour intervals until 96 hours of culture.

### Uptake of Bovine Serum Albumin

Uptake of fluorescent bovine serum albumin (FBSA) was assessed as previously described [36]. Briefly, confluent KSCs were cultured in serum-free medium for 24 hours, then incubated with 40 µg/ml Alexafluor® 594 conjugated BSA (Life Technologies) in PBS with CaCl_2_ and MgCl_2_ (Sigma) for 1 hour at 37°C. The samples were washed three times in PBS with CaCl_2_ and MgCl_2_, nuclei stained with Hoechst 33342 (Life Technologies), then fixed in 2% paraformaldehyde (PFA) (Sigma). The competitive inhibition of FBSA uptake was assayed as previously reported [37]. In brief, KSCs cultured for 24 hours in serum-free medium were incubated with 20 µg/ml FBSA, either alone or in the presence of either 500 ng/ml RAP (receptor associated protein) (R&D Systems) or 4 mg/ml BSA (Sigma) in PBS with CaCl_2_ and MgCl_2_ for 15 min at 37°C. The samples were washed once in PBS with CaCl_2_ and MgCl_2_, incubated with Hoechst 33342 for 4 min, then immediately fixed in 2% PFA and observed under a Leica DM2500 fluorescent microscope (Leica).

### Immunofluorescence Staining

Cultured cells were fixed in 4% PFA for 5 min at room temperature, and chimeric organoids were fixed in −20°C pre-chilled methanol for 7 min. The following primary antibodies were used: mouse anti-Wt1 (1∶500, Upstate), rabbit anti-Pax2 (1∶200, Covance), mouse anti-laminin-111 (1∶500, Sigma) mouse anti-megalin (1∶200, Acris Antibodies) mouse anti-synaptopodin (ready to use, Acris), mouse anti-calbindin-28 (1∶200, Abcam). The secondary antibodies used were: Alexa fluor-conjugated goat anti-mouse IgG1 (1∶500), Alexa fluor-conjugated chicken anti rabbit IgG (1∶500) and Alexa fluor-conjugated goat anti-rabbit IgG (1∶500) (all from Life Technologies). Nuclei were stained either with DAPI (Life Technologies), or Hoechst 33342 and observed under a Leica DM2500 fluorescent microscope. Chimeric organoids were always observed under a Leica AOBS SP2 confocal microscope (Leica).

### Chimeric Organ Culture

The chimeric kidney rudiment assay was performed as previously described [36] by recombining E11.5 re-aggregated mouse kidney rudiments with either QD-labeled (i) freshly isolated MM cells, (ii) 4 days cultured MM cells, or (iii) KSCs. For QD-labeling, MM cells or KSCs were labeled with 2.5 nM QTracker® 655 Cell Labeling kit (Life Technologies) following manufacturer’s instructions. Briefly, 2.5×10^5^ cells were incubated in suspension with 400 µl of labeling solution for 45 min at 37°C. Then the samples were washed three times in culture medium and used for chimera formation. Following labeling, a total of 2×10^4^ MM or KSCs were mixed with 6×10^4^ unlabeled E11.5 kidney rudiments cells, pelleted via centrifugation, and gently placed onto 1.2 µm Nucleopore filters in a Trowell-type culture system. Organoids were cultured for 2–5 days.

### RT-PCR

Total RNA was extracted from freshly isolated and cultured MM cells using TRIZOL (Life Technologies), following manufacturer’s instructions. A total of 200 ng of DNase-treated (Promega) RNA was reverse transcribed using random hexamers (Thermo Scientific) and Superscript III (Life Technologies). Primer pairs used for PCR reactions were as follows: *Wt1* 5′-CCAGTGTAAAACTTGTCAGCGA-3′, 5′-TGGGATGCTGGACTGTCT-3′; *Pax2* 5′-ACATCTGGTCTGGACTTTAAGAG-3′, 5′-GATAGGAAGGACGCTAAAGAC-3′; *Osr1* 5′-GCAGCGACCCTCACAGAC-3′, 5′-GCCATTCACTGCCTGAAGGA-3′; *Sall1* 5′-GCACATGGGAGGCCAGATCC-3′, 5′-GGAAGCGTCCGCTGACTTGG-3′; *Gdnf* 5′-GAAGTTATGGGATGTCGTGG-3′, 5′-GGATAATCTTCAGGCATATTGGAG-3′; *β-actin* 5′-GTTGACATCCGTAAAGACC-3′, 5′-CAGGAGGAGCAATGATCTTGA-3′. The cycling conditions were as follows: 1 cycle of 94°C for 5 min; 40 cycles of 94°C for 5 sec, 62°C for 30 sec, 72°C for 30 sec; 1 cycle of 72°C for 10 min. PCR products were sequenced at University of Dundee Sequencing Lab; furthermore, PCR products were resolved on 2.5% agarose gel and the band densities determined using ImageJ software.

### Quantitation of QD-labeled Cells in Chimeric Organoids

Following immunostaining, the percentage of freshly isolated MM, 4 days cultured MM cells and KSCs present within nascent nephrons was determined by counting the number of QD^+^ cells and the total number of cells within 7 randomly chosen developing nephrons (Wt1^+^ cells delimited by basement membrane) of chimeric organoids (n = 3 for each cell type). To determine whether the extent of integration into nascent nephrons was statistically significant among different cell types, one-way analysis of variance (ANOVA) and Student’s t-test were performed on samples with equal variance. Differences were considered statistically relevant when P<0.05.

## Results

### KSC-derived Proximal Tubule-like Cells Display Normal Absorptive Function

**Figure 1:**
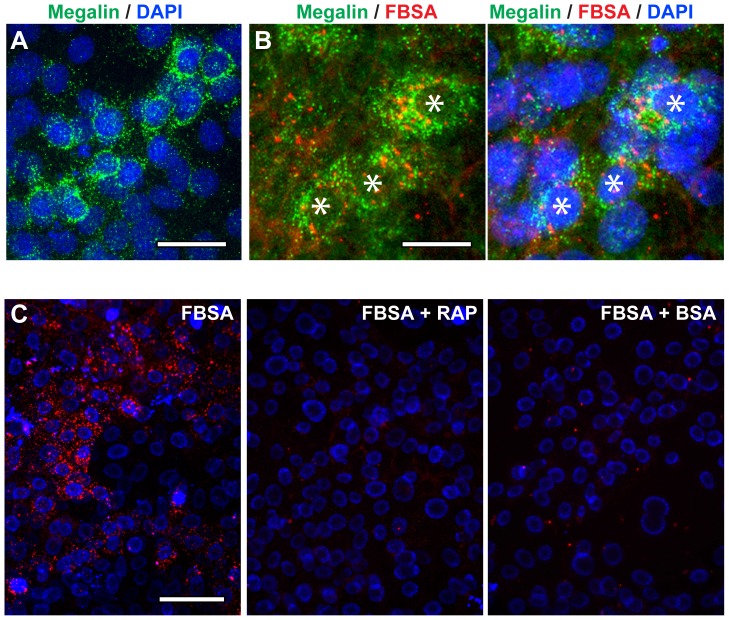
KSC-derived proximal tubule cells display normal absorptive function. (**A**) Immunofluorescence staining shows that some KSC-derived cells expressed the proximal tubule marker megalin (green). Nuclei are stained with DAPI (blue). (**B**) Immunostaining shows that FBSA (red) was uptaken by megalin^+^ KSCs (green); nuclei are stained with DAPI (blue). Asterisks indicate cells co-stained for megalin and FBSA. (**C**) *In vitro* functionality assay demonstrates that in the presence of either receptor-associated protein (RAP), or an excess of unlabeled BSA, the uptake of FBSA (red) was almost completely blocked. Nuclei are stained with Hoechst 33342 (blue). Scale bars are 50 µm (A), 25 µm (B) and 100 µm (C).

Previous studies have shown that stem/progenitor cells derived from mouse and human kidney can generate cells that express markers of proximal tubule cells [25], [26], [34] including megalin (Figure 1A), an endocytic receptor that plays a key role in mediating the reabsorption of proteins from the glomerular filtrate [38]. We have previously shown that KSCs derived from mouse neonates can spontaneously generate proximal tubule-like cells when cultured under routine conditions [34], but as yet, the functionality of these cells has not been demonstrated. To test the functionality of mouse KSC-derived megalin^+^ cells, we investigated their ability to internalize fluorescent bovine serum albumin (FBSA). Our results showed that cells within the population were able to internalize FBSA, as indicated by punctate cytoplasmic staining. Double immunofluorescence staining demonstrated that only cells expressing megalin displayed uptake of FBSA (Figure 1B), suggesting that FBSA uptake was mediated through the megalin receptor. To confirm this, the cells were incubated with FBSA in the presence of either excess unlabeled BSA, or receptor-associated protein (RAP), a megalin ligand that competitively blocks megalin-mediated protein uptake [37], [38]. Under both conditions, FBSA uptake was almost completely blocked, thus indicating that the uptake of BSA by KSC-derived proximal tubule cells is both saturable, and megalin-dependent (Figure 1C). These findings demonstrate that megalin^+^ KSC-derived proximal tubule-like cells display normal absorptive function *in vitro*.

### KSCs Integrate into Developing Kidneys *ex vivo* and Generate Proximal Tubule Cells and Podocytes that are Correctly Positioned within the Nascent Nephrons

**Figure 2:**
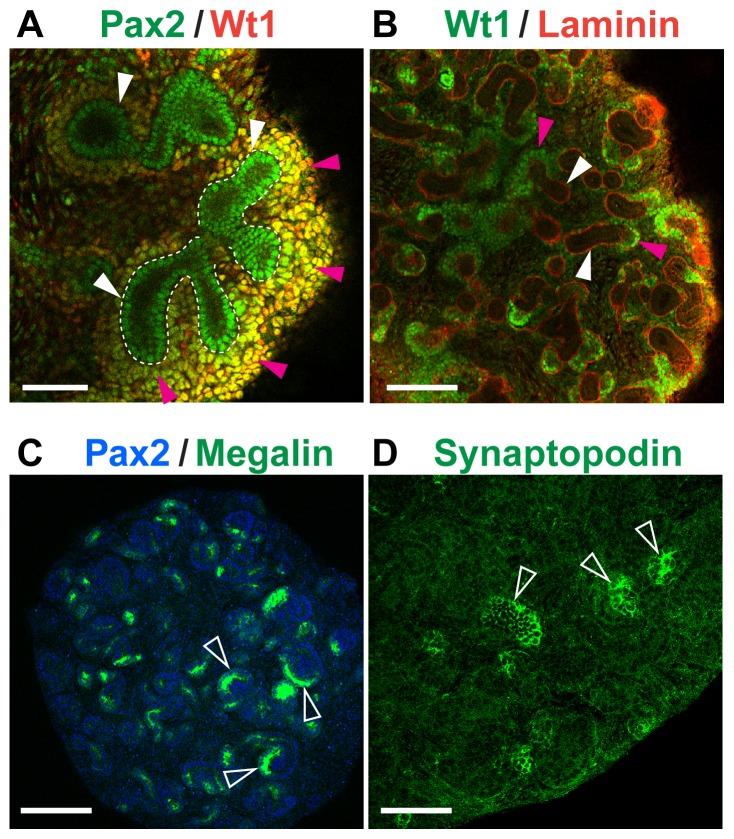
E11.5 re-aggregated kidneys form organotypic renal structures. (**A, B**) Immunostaining for Wt1 (red) and Pax2 (green) (A), or Wt1 (green) and laminin-111 (red) (B) shows the presence of condensed MM (pink arrowheads) surrounding the UB (white arrowheads) after 3 days of culture, thus demonstrating that the induction of the mesenchyme had taken place. (**C**) Immunostaining for megalin (green) and Pax2 (blue) at day 3 of rudiment culture reveals the presence of proximal tubules (open arrowheads). (**D**) Immunostaining for synaptopodin (green) demonstrates the presence of nascent podocytes within developing glomerular structures (open arrowheads) at 5 days of culture. Scale bars are 75 µm (A), 150 µm (B, C) and 100 µm (D).

**Figure 3:**
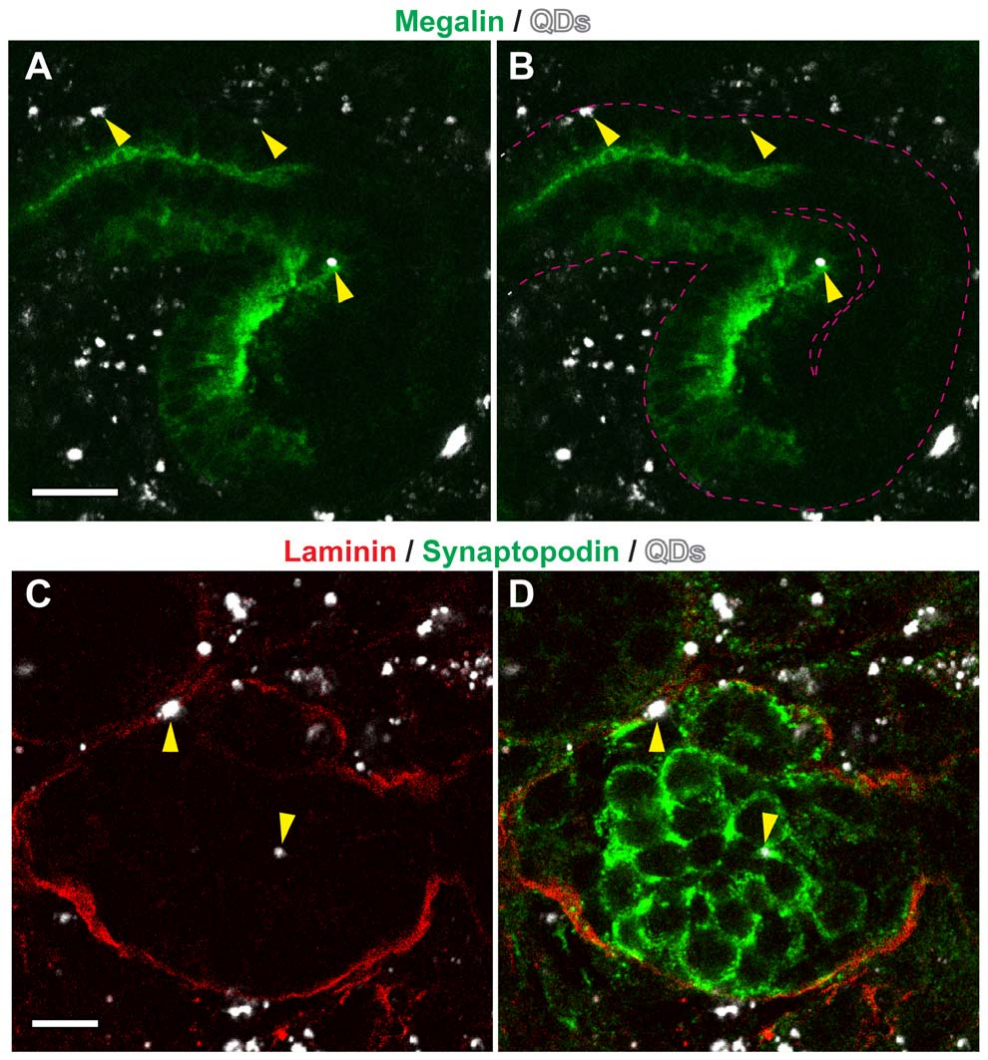
KSCs generate proximal tubule cells and podocytes within the developing nephrons. (**A**, **B**) Chimeric organoids were stained for megalin (green). Arrowheads indicate QD-labeled (white), megalin^+^ KSCs within a nascent proximal tubule. Note that megalin is present on the apical surface of the nascent proximal tubule (dotted lines in B), thus recapitulating its normal expression pattern observed *in vivo*. (**C**, **D**) Chimeric organoids were stained for synaptopodin (green) and laminin-111 (red). Arrowheads point to QD-labeled (white), synaptopodin^+^ KSCs within a nascent glomerulus. Scale bars are 20 µm (A, B) and 8 µm (C, D).

To investigate if the KSCs could integrate into developing kidney rudiments and give rise to renal cell types that were located appropriately within the nascent nephrons, we made use of an *ex vivo* culture system, in which E11.5 kidney rudiments are disaggregated into single cells, then re-aggregated to form organoids capable of developing renal-like structures [36]. After three days of culture, re-aggregated organoids contained regions of induced MM, as evidenced by the presence of Pax2^+^ Wt1^+^ cellular aggregates surrounding Pax2^+^ Wt1^−^ UB epithelium (Figure 2A, B). At the same time point, proximal tubule cells expressing apical megalin had also started to develop in the organoids (Figure 2C), and after five days, cells expressing the podocyte-specific marker, synaptopodin [39], could be observed within developing glomeruli (Figure 2D). This *ex vivo* culture system has previously been used to test the nephrogenic potential of various types of exogenous cells, such as embryonic stem cells (ESCs) [40], mesenchymal stem cells [41], and amniotic fluid stem cells [42] using a protocol that involves recombining the exogenous cell type under test with disaggregated kidney rudiments to generate a chimeric organoid. Using this system, chimeras were generated with the neonatal KSCs that had been labelled with quantum dots (QDs), fluorescent nanocrystals that are very effective for tracking cells over short time periods [43]. Following *ex vivo* culture, the chimeric rudiments were immunostained for megalin and synaptopodin. QD^+^ KSCs were found within both megalin^+^ proximal tubules and synaptopodin^+^ nascent glomeruli, indicating that the cells could differentiate to appropriately located renal cell types within the environment of the developing kidney (Figure 3).

### The Extent of KSC Integration into Developing Nephrons within Chimeric Rudiments is Similar to that of MM Cells

**Figure 4:**
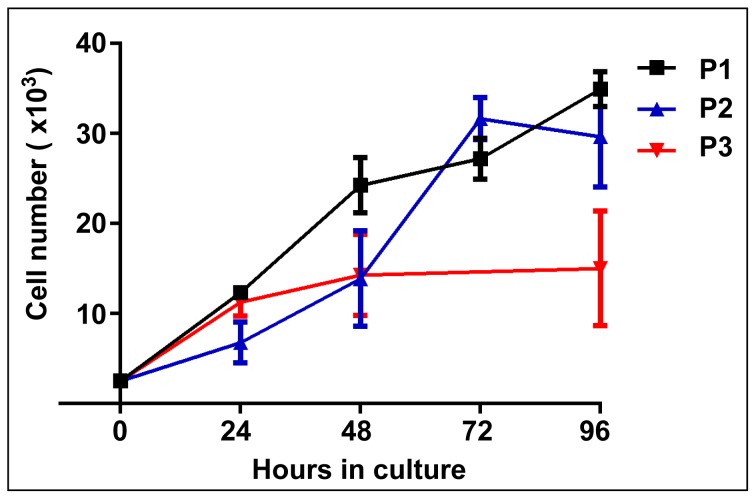
Population growth of cultured MM. Growth curve of MM cells cultured in the presence of Bmp7 and Fgf2 for the following times: days 1–4 (P1, black), days 5–8 (P2, blue), days 9–12 (P3, red). The MM cell population expanded by >12-fold during the P1 and P2 culture periods, but ceased expanding during P3. Data are expressed as mean ± SD; n = 6 for P1 and P2; n = 5 for P3.

**Figure 5:**
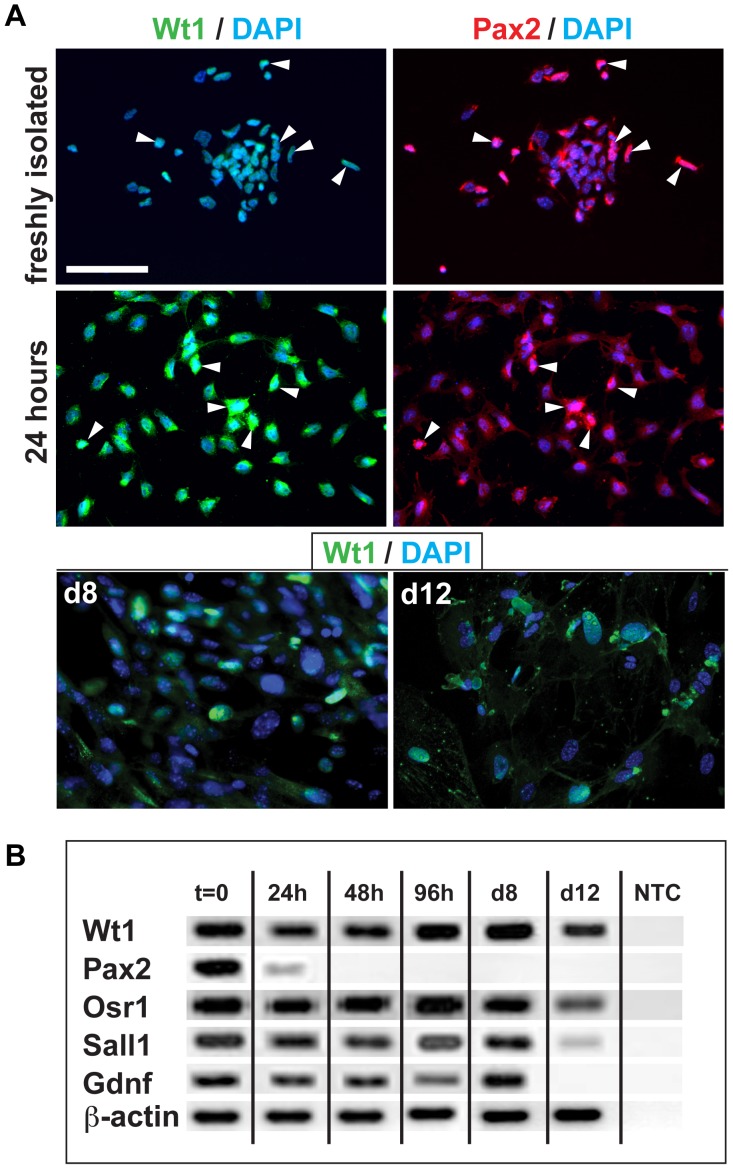
Expression of kidney progenitor markers in culture MM. (**A**) MM cells were cultured for the indicated time periods, and immunostained for Wt1 (green) and Pax2 (red). Wt1 was expressed in MM cells throughout the period studied, but there were noticeably fewer Wt1^+^ cells by day 8. Pax2 was only detected for the initial 24 hours of culture. Nuclei were stained with DAPI (blue). Scale bars for all images, 100 µm. (**B**) RT-PCR shows the expression profile of key MM markers during the 12-day culture period. NTC, no template control.

Using Wt1-Cre/Rosa26R mice, we have previously shown that the mouse neonatal KSCs used here are derived from the MM and express several MM markers [34]. Given these similarities between KSCs and MM cells, we next investigated if the KSCs could integrate into developing nephrons to a similar extent as MM cells. To counter the possibility that any difference in KSC and MM integration potential could be due to the KSCs having been cultured extensively *in vitro*, we first established a population of cultured MM cells so that the KSCs could be compared with both freshly isolated and cultured MM. To this end, MM cells were isolated from E11.5 embryos and cultured in the presence of Fgf2 and Bmp7, which have been shown to support the survival and growth of MM in the short-term [14], [15]. As expected, in the absence of Bmp7 and Fgf2, the MM failed to grow (data not shown), whereas in the presence of these growth factors, the MM population expanded >12-fold during both the first (P1) and second (P2) 4 day culture periods. Population growth during the third culture period (P3) was limited, with no further growth occurring after day 10 (Figure 4). Thus, the growth kinetics of cultured KSCs and MM cells are very different, with KSCs displaying unlimited self-renewal [34], and MM cells ceasing to grow following 10 days of *in vitro* culture. To investigate if the expression of key MM markers was maintained during the *in vitro* culture period, immunostaining for Wt1 and Pax2, and semi-quantitative RT-PCR for *Wt1*, *Pax2*, *Osr1*, *Sall1* and *Gdnf* were performed. MM cells expressed Wt1 throughout the culture period, although from day 8, there were noticeably fewer Wt1^+^ cells (Figure 5A, B, S1). Pax2 protein was detected in some cells during the initial 24-hour culture period, but neither Pax2 protein (not shown) nor mRNA were detectable from 48 h onwards (Fig. 5A, B, S1). In contrast, expression of *Wt1*, *Osr1*, *Sall1* and *Gdnf* mRNA was maintained over the first 8 days of *in vitro* culture, and did not decrease until the third culture period (days 8–12) (Figure 5B, S1).

**Figure 6:**
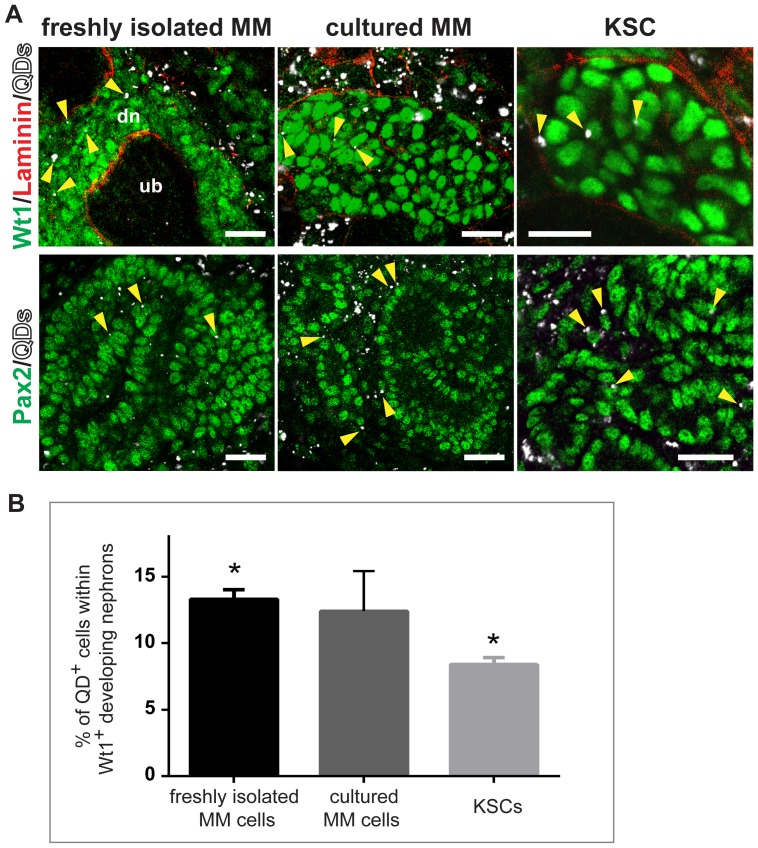
The extent of KSC integration into developing nephrons within chimeric rudiments is similar to that of MM cells. (**A**) Chimeras generated with QD+ (white)-labelled (i) freshly isolated MM, (ii) MM that had been cultured for 4 days, or (iii) KSCs, were cultured for 3 days and immunostained for Wt1 (green) and laminin-111 (red), or for Pax2 (green). Arrowheads point to QD+ cells integrated within the forming nephrons. Scale bars are 30 µm, 15 µm and 15 µm (upper row), and 36 µm, 31 µm and 10 µm (lower row). (**B**) The percentage of KSCs that integrated into developing nephrons was significantly lower than that of freshly isolated MM cells, but not statistically different to that of MM cells cultured for 4 days (Student’s *t*-test; P>0.05). Results are expressed as mean ± SE. n = 3 in each group; for each organoid, 7 random developing nephrons were selected for analysis.

We next compared the ability of KSCs and *in vitro* cultured MM cells to integrate into developing nephrons using the kidney rudiment assay. For this purpose, both cell types were labelled with QDs and individually recombined with disaggregated E11.5 kidney rudiments. Following a 3-day culture period, the chimeric organoids were immunostained for Pax2, Wt1 and laminin to visualise developing renal structures. Chimeric organoids containing freshly isolated MM served as positive controls. The integration pattern of QD^+^ cells was similar in all three types of chimeras, as labelled cells were observed within Wt1^+^ aggregates and Pax2^+^ nephron tubules, but rarely detected within the UB (Figure 6A). To compare the extent of integration, the proportion of QD^+^ cells within developing nephrons was determined for the three types of chimeric organoid, as we have previously undertaken [40]. The percentage of QD^+^ freshly isolated MM cells, *in vitro* cultured MM cells and KSCs that integrated into developing nephrons was approximately 13%, 12% and 9%, respectively. Statistical analyses indicated that the extent of cultured MM cell integration was not significantly different than that observed with freshly isolated MM cells or KSCs, but the extent of KSC integration was significantly less than that of freshly isolated MM cells (Figure 6B). These data show that although the KSCs and MM cells are derived from different life stages (i.e., neonatal compared with embryonic), and display very different growth kinetics in culture, their integration behaviour within chimeric kidney rudiments *ex vivo* is strikingly similar.

## Discussion

In this study we have shown that mouse neonatal KSCs can generate proximal tubule-like cells *in vitro* that display megalin-dependent absorptive function. Furthermore, when stimulated by induction processes within kidney rudiment chimeras *ex vivo,* the KSCs give rise to proximal tubule cells and podocytes that are correctly positioned within the nascent nephrons. On comparing the ability of MM cells and KSCs to integrate into developing kidneys *ex vivo*, we found that freshly isolated MM cells, cultured MM cells and KSCs integrate to a similar extent into nascent nephrons but are excluded from UB branches.

We have recently shown that the neonatal KSCs used here are derived from the MM, express several stem cell and MM-specific markers, and can spontaneously generate podocyte- and proximal (but not distal) tubule-like cells in culture [34]. In comparison, MM cells have a broader differentiation potential which allows them to generate all cells of the nephron. However, the MM cells are unable to spontaneously give rise to these cells in culture, requiring co-culture with an inducer [7]–[11]. KSCs and MM cells also differ in their proliferation kinetics, since KSCs display unlimited self-renewal [34], whereas MM cells undergo a limited number of cell division [16], [20]. Thus, although it is generally thought that stem cells differentiate to shorter-lived progenitor cells, which ultimately differentiate to specialised cells, it appears that in the developing kidney, the reverse situation applies, with the transient population of MM progenitor cells giving rise to one or more stem cell populations. However, it is not clear if such tissue-specific adult stem cells really exist *in vivo* or are simply tissue culture artefacts [44]. In support of the existence of KSCs *in vivo*, a bipotential stem cell capable of generating podocytes and proximal tubule cells has been identified within the Bowman’s capsule of the adult human kidney [26]. More recently, it has been shown that these human kidney-derived stem cells can generate proximal tubule cells following intravenous administration into mice with induced tubular injury [31]. However, renal differentiation was evaluated solely on the basis of marker expression, and the functionality of the stem cell-derived renal cells was not addressed. In the current study, we demonstrate that proximal tubule-like cells derived from neonatal mouse KSCs expressing alkaline phosphatase, aquaporin and megalin [33], also display megalin-dependent protein uptake *in vitro*. An important role of megalin^+^ proximal tubule cells *in vivo* is to endocytose macromolecules from the glomerular filtrate [45]. Thus, postnatal mammalian KSCs are indeed able to generate proximal tubule-like cells that display functionality.

The postnatal KSCs used in this study appear to share some properties with a stem cell population derived from E12.5 mouse kidneys, as both can self-renew, are clonogenic, can integrate into mouse kidney rudiments, and can generate non-renal cell types, including adipocytes and osteoblasts [34], [46]. However, in contrast to the neonatal KSCs described here, the stem cell population derived from E12.5 kidneys did not appear to generate any *megalin*-expressing cells, suggesting that they were unable to differentiate to proximal tubule cells; the ability of the E12.5 kidney-derived stem cells to generate podocytes was not investigated in the previous study [46].

A necessary part of our study was to establish a population of cultured MM cells so that the extent of integration of the *in vitro*-derived KSCs into developing nephrons could be compared with both freshly isolated and *in vitro* cultured MM. Consistent with two earlier studies [11], [14], we found that cultured MM cells rapidly lost Pax2 expression. Strikingly, we found that if these MM cells were placed within the environment of the embryonic kidney, they could re-express Pax2 and participate in the development of nephron tubules. These results show that the environment of the developing kidney has a strong inductive effect on cultured MM cells. This is in line with recent studies where even some non-renal cell types, such as embryonic stem cell-derived mesoderm [40] and amniotic fluid stem cells [42] could be induced to adopt a renal fate when placed in kidney rudiment chimeras. In the present study, we found that freshly isolated MM, cultured MM and KSCs were rarely present within the UB, but integrated into nascent nephrons to a comparable extent. Thus, although the KSCs were derived from postnatal kidneys, when placed within the environment of the mouse embryo kidney, their behavior appeared similar to that of the MM.

Using the same chimeric kidney organoid system described in this study, we have recently shown that mouse ESC-derived mesoderm could integrate into developing nephron structures and generate proximal tubule cells and podocytes [40]. Interestingly, the ESC-derived mesoderm comprised 12–13% of the developing nephron structures, suggesting that uncommitted nascent mesoderm is primed to respond to the environment of the embryonic kidney and generate renal cell types as efficiently as the nephron progenitors themselves. However, while MM cells and KSCs in the chimeric rudiment assay could only give rise to nephron structures, the ESC-derived mesoderm, which was not committed to a renal fate, could generate both nephron and UB cells. This supports the notion that mesoderm has a broad differentiation capacity while MM cells and KSCs are restricted to the MM-derived lineage.

It is important to note that the KSCs used in this study were derived from mice within the first week of postnatal life [34], when nephrogenesis is still ongoing. It is therefore unclear if the KSC derived from the progenitor cell population are still involved in normal neonatal kidney development, or alternatively, if they arise from an unidentified cell population that remains in the kidney after its development is complete. Future studies will address this question by investigating if stem cells with properties similar to the neonatal KSCs used here can also be isolated from adult mouse kidneys.

## Supporting Information

Figure S1 **Gene expression analysis of freshly isolated and cultured MM cells.** The histograms show the expression levels of the indicated MM markers in freshly isolated MM and in MM cells cultured for different periods of time, as indicated. The results were normalized to the expression levels of β-actin.
(PDF)
