# Correction: Stem Cells Derived from Neonatal Mouse Kidney Generate Functional Proximal Tubule-Like Cells and Integrate into Developing Nephrons *In Vitro*

**DOI:** 10.1371/annotation/57bd3cdf-4d63-41a6-972a-904e00c369ad

**Published:** 2013-06-28

**Authors:** Egon Ranghini, Cristina Fuente Mora, David Edgar, Simon E. Kenny, Patricia Murray, Bettina Wilm

The name of the second author was given incorrectly in the Citation. 

The correct citation is: Ranghini E, Fuente Mora C, Edgar D, Kenny SE, Murray P, et al. (2013) Stem Cells Derived from Neonatal Mouse Kidney Generate Functional Proximal Tubule-Like Cells and Integrate into Developing Nephrons In Vitro. PLoS ONE 8(5): e62953. doi:10.1371/journal.pone.0062953. 

