# Supplementary figures and images for: Stem Cells Derived from Neonatal Mouse Kidney Generate Functional Proximal Tubule-Like Cells and Integrate into Developing Nephrons In Vitro
Source: PLoS One. 2013 May 7;8(5):e62953. doi: 10.1371/journal.pone.0062953 (PMC3646983; doi:10.1371/journal.pone.0062953)

Figure S1

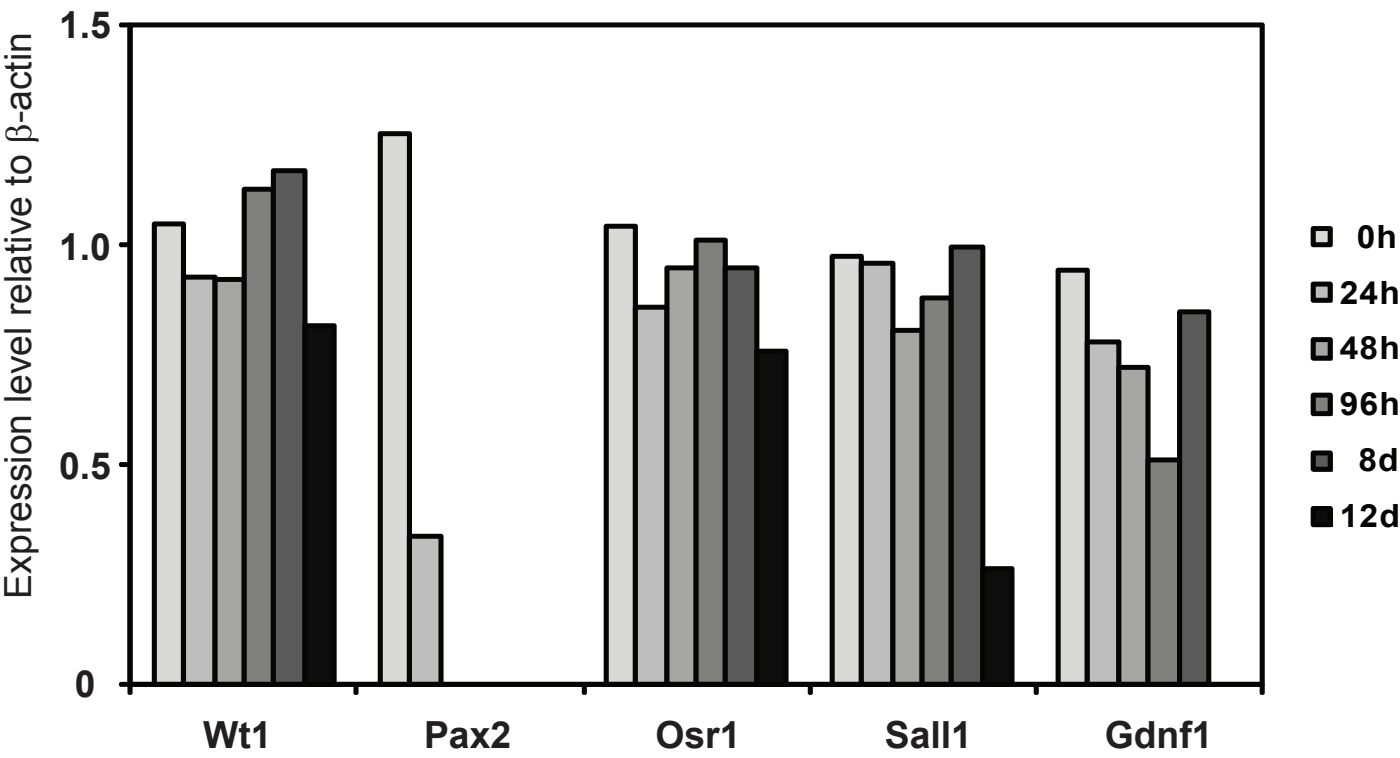

Supplement: Figure S1 — Gene expression analysis of freshly isolated and cultured MM cells. The histograms show the expression levels of the indicated MM markers in freshly isolated MM and in MM cells cultured for different periods of time, as indicated. The results were normalized to the expression levels of β-actin. (PDF) [file pone.0062953.s001.pdf]
